# Glucagon-Like Peptide-1 Agonists and General Anesthesia: Perioperative Considerations and the Utility of Gastric Ultrasound

**DOI:** 10.7759/cureus.58042

**Published:** 2024-04-11

**Authors:** Conner M Willson, Love Patel, Peter Middleton, Mihir Desai

**Affiliations:** 1 Department of Clinical Medicine, Des Moines University, Des Moines, USA; 2 Department of Anesthesiology, University of Florida College of Medicine – Jacksonville, Jacksonville, USA

**Keywords:** pulmonary aspiration, gastric ultrasound, gastric emptying, general anesthesia, glp-1 agonists

## Abstract

Glucagon-like peptide-1 (GLP-1) agonists are very popular and useful medications for the treatment of type 2 diabetes mellitus and obesity. Potent gastric emptying delay is common with these medications, serving as a major contributor to the postprandial glycemic control and weight loss benefits of these medications. Recently, multiple case reports and studies indicating safety risks for these medications and their use in patients planning to undergo general anesthesia have been published, as retained gastric contents can lead to intraoperative aspiration. New guidelines for these medications have been released to guide clinical practice for anesthesiologists. Some degree of preoperative cessation of these medications is required. At this time, the ideal window for cessation of these medications to optimize clinical efficacy while reducing aspiration risks has not yet been well elaborated on. Aspiration of gastric contents can still occur despite appropriate preoperative fasting in patients taking GLP-1 agonists. Gastric ultrasound appears to be an effective and objective way of preoperatively assessing a patient’s stomach contents to make decisions regarding anesthetic management for patients prescribed these medications. This practice is limited by a general lack of training and implementation in current anesthesiology practice.

## Introduction and background

Over the past few years, no medication class has been nearly as popular in the media as glucagon-like peptide-1 (GLP-1) agonists. Among this class, semaglutide, better known by the trade names Ozempic and Wegovy, has been most notable for making headlines for its impressive weight reduction and appetite control effects. These medications are designed to be similar to the endogenous incretin GLP-1 but with longer-lasting and more potent effects. Among these effects are significant changes in gastric motility. Slowing gastric motility is very beneficial for patients with obesity and type 2 diabetes mellitus (T2DM), as this encourages a reduction of food intake. However, while very beneficial in this realm, the consequences of these medications with regard to general anesthesia were not well understood prior to their Food and Drug Administration (FDA) approval. Numerous recent perioperative adverse events have been documented in patients utilizing GLP-1 agonists, prompting the American Society of Anesthesiologists (ASA) to release new guidelines regarding these medications [1].

GLP-1 agonists exert their effects by mimicking the endogenous hormone GLP-1, which is secreted physiologically by enteroendocrine “L-cells” of the small and large intestines in response to the consumption of a meal [2-3]. Another important incretin is the hormone glucose-dependent insulinotropic peptide (GIP), which possesses similar effects. The major difference among these incretins is the effect of T2DM on receptor sensitivity. Unlike GLP-1, GIP receptors undergo desensitization in T2DM patients [4]. Despite this, tirzepatide, a newer medication in this class, has dual GIP and GLP-1 receptor affinity. All other FDA-approved GLP-1 agonists only exert effects on the GLP-1 receptor [5]. Both hormones in their endogenous forms are rapidly metabolized after release via the enzyme dipeptidyl peptidase-4 (DPP-4) [6]. Exogenous GLP-1 agonists are designed to be resistant to DPP-4 degradation, leading to prolonged half-lives. Furthermore, the bioavailable doses of these medications are significantly larger than those of typical endogenous release [3].

GLP-1 is physiologically released in two distinct phases: first, within minutes of eating, followed by a second release up to one hour later [2-3]. Activation of the GLP-1 receptor results in the activation of stimulatory G-protein coupled receptors on pancreatic β-cells, increasing insulin secretion. GLP-1 has inhibitory effects on pancreatic α-cells, leading to a reduction in glucagon secretion [2-3]. Most notably, from an anesthesia perspective, GLP-1 causes a vagal-mediated reduction in gastric motility and acid secretion [7]. Furthermore, GLP-1 has been shown to increase the secretion of somatostatin from pancreatic δ-cells [4]. Somatostatin further functions to reduce gastric motility and pancreatic secretions [8].

Given the pharmacology of these agents, the positive benefits for patients with obesity and T2DM are strong. However, these effects have proved to be worrisome for anesthesiologists, as loss of gastric motility poses a higher risk of pulmonary aspiration [9]. These potential consequences have been highlighted in recent articles and case reports [10-12]. This review is set out to consolidate the current literature regarding this topic.

## Review

Perioperative risks

Numerous prior studies have confirmed the theoretical effects of GLP-1 agonists on gastric motility via the use of nuclear scintigraphy [13-16]. Recent clinical studies have identified a higher proportion of retained gastric content in patients taking GLP-1 agonists, notably semaglutide. A retrospective cohort study determined that semaglutide use was associated with a significantly higher prevalence ratio for increased residual gastric contents compared to control. All patients underwent appropriate preoperative fasting based on new ASA guidelines, but there was no preoperative cessation of their GLP-1 agonist. This study additionally found a higher prevalence ratio associated with the presence of preoperative digestive symptoms, such as bloating or a feeling of fullness. This effect appeared to be synergistic with semaglutide use. This study did have one total episode of pulmonary aspiration in total, occurring in the GLP-1 agonist group [17].

A case-control study from March 2023 found that there was a significant positive correlation with the presence of increased retained gastric contents, determined by esophagogastroduodenoscopy (EGD), in patients on GLP-1 agonists compared to control (5.4% to 0.5%). This effect was seen across multiple types of long-acting GLP-1 agonists but was not found to occur with DPP-4 inhibitors [17].

Numerous case reports of adverse events with GLP-1 agonists have been published over the last two years. One case report detailed an episode of aspiration in a patient on semaglutide, despite appropriate preoperative fasting [10]. Another case report details a patient on semaglutide with a large volume post-induction regurgitation of gastric contents, fortunately without evidence of aspiration [11]. A third case report detailed large amounts of gastric contents discovered via nasogastric tube suction after uneventful induction and intubation in a patient who recently began tirzepatide and was additionally on chronic opioids. This patient had intraoperative emesis containing solid contents, which she later identified as food consumed “several days” prior to her procedure [12]. In all case reports reviewed, every patient underwent adequate preoperative fasting; however, there was no preoperative cessation of their GLP-1 agonist.

Some literature was found that contains conflicting data for the aforementioned studies. A 2020 meta-analysis concluded that the benefits of perioperative continuation outweighed the potential risks of discontinuation of these medications [18]. However, the risks of delayed gastric emptying and general anesthesia were notably not discussed. This meta-analysis was based on earlier studies, prior to the recent influx of cases the medical community has seen since GLP-1 agonists exploded in popularity. Another contradictory 2022 retrospective cohort study found that there was a non-significant increase in retained gastric contents in patients undergoing an EGD who were taking a GLP-1 agonist [19].

As mentioned earlier, patients who have concomitant gastric symptoms in addition to GLP-1 use are at much higher risk of aspiration. Furthermore, known risk factors for aspiration and delayed gastrointestinal motility must be considered in patients taking GLP-1 agonists who are scheduled to undergo general anesthesia. Factors such as opioid use, known diabetic gastroparesis, gastrointestinal anatomical or post-surgical changes, and a lack of appropriate preoperative fasting most likely significantly worsen this risk of aspiration if present alongside GLP-1 agonist use. Clinicians must be cognizant to evaluate the patient’s medical history and medication list and to query regarding symptoms in the immediate preoperative period, especially for patients with a history of GLP-1 agonist use.

Beneficial perioperative effects of GLP-1 agonists and risks of cessation

GLP-1 agonists present plenty of potential positive perioperative effects. As these medications have been shown to be effective adjuncts in the treatment of T2DM, cessation may present concerns for reduced blood glucose control. Perioperative hyperglycemia poses an increased risk for surgical site infection and perioperative mortality [20]. GLP-1 agonists, on average, cause a hemoglobin A1c reduction of 0.5-1% [3]. The degree of glucose control regulated by GLP-1-mediated insulin release versus appetite control effects is not conclusive. It is reasonable to assume that appropriate preoperative fasting occurs in all elective surgeries where temporary drug cessation is considered. Therefore, glucose dysregulation in the postoperative period will be solely related to the lack of endocrine effects from GLP-1 agonists in patients who temporarily pause these medications. While this is an important consideration, there is no literature at this time to help delineate whether or not this is a significant enough factor to consider when compared to the aspiration risk that GLP-1 agonists provide.

In addition to hypoglycemic effects, these medications appear to offer some reduction in major adverse cardiac event (MACE) occurrence [21]. At the time of writing, the effect of GLP-1 agonists specifically on postoperative MACE has not been studied extensively. This is a minor benefit that is unlikely to outweigh the consequences of aspiration for most patients.

Appropriately timing surgery around the use of these medications is difficult and complex. The optimal window for a temporary hold is currently unknown. Despite the presence of daily-dosed forms, the most common GLP-1 agonists used at this time are weekly injectable medications. As a result of evading DPP-4 degradation, these agents possess very long half-lives [2-3]. As a result, it is unreasonable to expect patients to hold onto the use of these medications long enough to achieve negligible plasma levels, as this could take up to five weeks.

Another element of consideration regarding these medications is rapid-onset tachyphylaxis, which has been documented in the past. Effects on vagal inhibition can be reduced significantly as early as the second dose of a GLP-1 agonist [22]. Although tachyphylaxis is a known phenomenon with these medications, more research is necessary to understand what patient-specific factors may influence this process and how this phenomenon will influence surgical planning. At this time, undergoing general anesthesia near the initiation of these medications should be avoided if possible. The initiation period is likely when the most potent gastric emptying effects exist. In addition, discussions regarding patient adherence to their treatment plan are necessary given recent shortages of GLP-1 agonist medications, such as semaglutide. A break and restart in GLP-1 agonist use near the time of surgery could theoretically present a significantly higher risk of aspiration compared to uninterrupted use.

These tachyphylaxis effects have consequences for missing doses, as is now indicated preoperatively per ASA guidelines. Patients restarting these medications may experience more potent GI side effects than they were previously accustomed to [23]. Therefore, surgeons and anesthesiologists must be diligent in discussing expectations for the postoperative period. Patients can be referred to the missed dose recommendations of their specific type of GLP-1 agonist. Some GLP-1 agonists require restarting the initial uptitration process of the medication if a long enough time period has passed since the last dose. In these scenarios, patients may additionally experience a reduction in efficacy and less predictable blood glucose levels until their previous dose steady-state is achieved [23].

There is great variety amongst the class of GLP-1 agonists regarding their pharmacokinetic properties (Table 1). Clinicians should be aware of some of these characteristics, such as the extraordinarily long half-lives of dulaglutide, semaglutide, and tirzepatide. General anesthesia should be avoided if possible during the time of peak concentration for these medications, as this theoretically presents the highest risk of aspiration [24-29].

**Table 1:** List of GLP-1 agonists approved in the United States and their relative approved indication, dosage, time to maximum concentration, and half-life FDA: Food and Drug Administration; ER: extended-release; IR: immediate-release; GLP-1: glucagon-like peptide 1; GIP: glucose-dependent insulinotropic peptide; T2DM: type 2 diabetes mellitus; SQ: subcutaneous; PO: oral; ASA: American Society of Anesthesiologists Based on the ASA chart published with guidelines article on GLP-1 use [1], with the simplified format and other modifications [24-29].

Drug Name (Brand)	Agonism	FDA Approval	Dosing Instructions	Time to Maximum Concentration	Half-life (t_1/2_)
Dulaglutide (Trulicity)	GLP-1	T2DM	SQ, weekly	24-72 hours	120 hours
Exenatide ER (Bydureon); exenatide IR (Byetta)	GLP-1	T2DM, obesity	SQ, weekly (ER); SQ, twice daily (IR)	4-8 hours (ER); 2 hours (IR)	2.4 hours
Liraglutide (Saxenda, Victoza)	GLP-1	T2DM, obesity	SQ, daily	8-12 hours	13 hours
Lixisenatide (Adlyxin)	GLP-1	T2DM	SQ, daily	1-2 hours	3 hours
Semaglutide SQ (Ozempic, Wegovy); semaglutide PO (Rybelsus)	GLP-1	T2DM, obesity	SQ, weekly; PO, daily	24-72 hours (SQ); 1 hour (PO)	168 hours
Tirzepatide (Mounjaro, Zepbound)	GLP-1 and GIP	T2DM, obesity	SQ, weekly	24-48 hours	120 hours

In total, GLP-1 agonists do present some minor perioperative benefits, but none important enough to outweigh the risk of serious consequences of aspiration. GLP-1 agonists have long half-lives that reasonably prevent reaching negligible plasma levels. General anesthesia must be avoided in the acute period after starting these medications (first few doses), and a discussion regarding the reintroduction process must take place with patients using GLP-1 agonists.

Gastric ultrasound

Point-of-care gastric ultrasound is a quick and inexpensive way of determining a patient’s need for increased precautions prior to undergoing general anesthesia. This technique has been shown to be very sensitive and specific for determining if a patient has a full stomach and is at high risk of intraoperative aspiration. Patients with a high clear gastric liquid volume or any solid contents in the stomach should either be treated with “full stomach” precautions or have their case delayed, depending on necessity and clinical picture [30-31].

**Figure 1:**
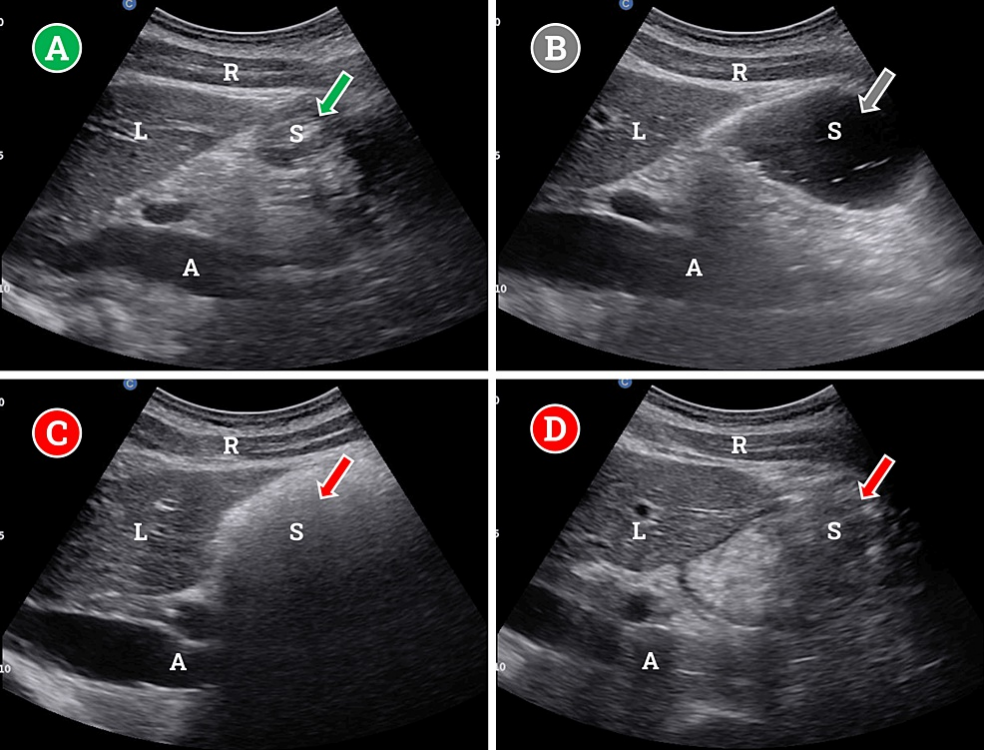
Gastric ultrasound findings based on different types of gastric contents. (A) Target-like appearance of the empty stomach (flat antrum), low risk of aspiration; (B) dilated stomach with liquid contents, volume assessment required; (C) stomach with solid contents and air creating artifact, early phase following eating, high risk of aspiration; (D) dilated stomach with hypoechoic components, later phase following solid or non-clear liquid consumption. R: rectus abdominis muscle; L: liver; S: stomach (antrum); A: aorta Images were taken and generated by the authors for the purpose of publishing in this journal only.

The interpretation of gastric sonography is relatively simple. An empty stomach will appear target-like at the antrum, a small, anechoic center surrounded by hypoechoic walls (Figure 1A). With this finding, the risk of aspiration is very low. A stomach with clear fluid content will appear distended and anechoic throughout (Figure 1B). This finding requires volume evaluation to guide decision-making.

Higher-risk contents like thick liquids (such as milk) and solids present a high risk of aspiration. Early after consumption, solids are easily detectable, creating significant sonographic artifacts that obscure deeper structures (pancreas, aorta) due to the introduction of air into the stomach (Figure 1C). Later, after consumption, solids and thicker liquids will appear hyperechoic in a distended stomach, distinct from that of clear liquids (Figure 1D).

The primary gray area for decision-making comes down to the interpretation of clear gastric contents (Figure 1). A single measurement of the cross-sectional area at the gastric antrum with the patient in a right lateral decubitus position is effective. While there is no universal consensus on the volume of clear stomach contents, prior studies have utilized a cutoff of ≤1.5 mL/kg of liquid in the stomach, or approximately 100-130 mL, as evidence of an empty stomach at low risk of aspiration [32-34]. While multiple methods to quantify gastric volume based on ultrasonography have been developed, the method developed by Perlas et al. is based on RLD positioning, was validated based on endoscopy, and allows for a rough estimate based on the age of the patient and the cross-sectional area of the gastric antrum. The calculation is as follows [33,35]:

Gastric volume (mL) = 27.0 + 14.6 * (cross-sectional area in cm2) - 1.28 * (patient age)

As we believe gastric ultrasound is an invaluable tool in the landscape of high GLP-1 agonist use, we recommend utilizing a qualitative approach first to differentiate between high- and low-risk findings (Figure 1). For clear gastric contents with a need for volume assessment, we recommend clinicians utilize the quick reference provided by Perlas et al. to reduce the need for calculation [35].

A recent study conducted using non-obese participants taking semaglutide demonstrated that gastric ultrasound was effective in visualizing solid gastric contents after an eight-hour fast, especially in the right lateral decubitus position [36]. Not only does this study add weight to the utility of gastric ultrasound in the preoperative evaluation of GLP-1 agonists, but it also presents more confirmatory evidence that semaglutide poses a significant risk for aspiration. On a separate note, gastric ultrasound has been successfully utilized in the past to guide the anesthetic management of patients who did not follow fasting instructions [34]. Another study revealed that in patients with GLP-1 agonists set to undergo general anesthesia, gastric ultrasound revealed a higher burden of retained gastric contents, often even in the setting of the ASA-recommended preoperative cessation of these medications [37]. This study further elucidated that the duration of medication cessation was not predictive of increased retained gastric contents. This is evidence that these medications create an unpredictable timeline for gastric emptying, and therefore, a screening modality such as gastric ultrasound may be required to better assess a patient’s risk of pulmonary aspiration at the time of evaluation in the preoperative period.

Overall, point-of-care gastric ultrasound presents an easily accessible and simple solution to evaluating patients utilizing GLP-1 agonists. Clinicians should look to practice gastric ultrasound and incorporate it into their preoperative evaluations for these patients. Further investigation into this field will help to elaborate just how practical and effective this study is, as well as to develop concrete standards for indications for screening and cutoff values for clear stomach contents.

Future investigation and discussion of the current landscape

Discovering the optimal timing for the cessation of GLP-1 agonists is of utmost importance. To reduce perioperative risks and guarantee glycemic control, researchers should investigate the best window of time for stopping GLP-1 receptor agonists prior to surgery. Establishing reliable recommendations for discontinuation timing will benefit from research into the length of GLP-1 agonist effects, their half-lives, and how they interact with patient-specific variables. By answering these concerns, perioperative treatment options for GLP-1 agonist users will be improved, thereby enhancing patient safety and medical procedures. Unfortunately, given the half-lives of the most commonly used GLP-1 agonist medications [24-29] and evidence of the unpredictability of gastric emptying in patients using these medications [17,37], gastric ultrasound may be a necessary tool to identify patients at high risk of aspiration in the preoperative period.

There is a great likelihood that patient use of GLP-1 agonist medication will only continue to increase [38]. Discussion regarding the utility of these medications continues to move rapidly, and it is likely that indications for GLP-1 agonists will expand significantly in the coming years. Currently, research is consistently being published on conditions that affect large portions of the population, such as substance use disorder, metabolic dysfunction-associated steatotic liver disease, polycystic ovarian syndrome, and numerous other conditions [39]. On another note, as these medications only exist in their brand-name formulations, financial barriers prohibit their use by a large number of patients who are prescribed them. As some GLP-1 agonist medications near patent expiration in the coming years, we can expect generic formulations to increase use dramatically as well [40]. This evidence of expanding use undoubtedly signals a need to further investigate the intricacies of these medications and how they may affect anesthetic management.

Compounding pharmacy-based formulations of these medications have become popular and controversial in the past year, notably for semaglutide. Interest in this practice arose because of financial barriers and supply shortages. These formulations are more likely to have improper concentrations of the medication or utilize unknown processes and adjuvants to create a similar effect [23,41]. Major concerns arise via the administration of devices for subcutaneous injectables. A prior case series raised concerns regarding inappropriate dosing stemming from a lack of patient education and appropriate instructions, coupled with non-standard injection equipment [42]. We suspect that this could additionally lead to errors in patients reporting the use of GLP-1 agonists in the preoperative period. Patients may receive injections directly from ancillary facilities, such as weight loss or aesthetic centers [42]. They may obtain the medication from outside the country or from online pharmacies [41]. Regardless, they will receive their medications outside of a standard pharmacy. For both reasons, they may view their GLP-1 agonist differently than their standard medications and more like a supplement. In addition, all processes remove necessary regulatory checkpoints, and multiple opportunities for error arise. Discussion regarding the use of these medications may be beneficial in preoperative discussions with every patient. Patients on non-standard formulations of GLP-1 agonists may need individualized and detailed instructions for cessation. Anesthetic management of patients utilizing compounded semaglutide presents a unique and largely uncontrollable problem. For patient safety reasons, we hope that these forms of GLP-1 agonists become less and less utilized as time moves on.

Advances in point-of-care testing, such as gastric ultrasonography, will be incredibly beneficial in mitigating the possible negative consequences of these medications perioperatively. Further research is required to examine the availability, use, and accuracy of point-of-care gastric ultrasonography. To further improve patient safety, it will be very helpful to understand the barriers to its general adoption into clinical practice and to investigate new technologies for determining stomach volume.

## Conclusions

GLP-1 agonists are revolutionary medications that continue to be utilized by an increasing number of patients. They exert very potent gastric slowing effects that can cause rare yet severe complications with general anesthesia. Recent adverse events and larger studies have initiated the ASA to release new recommendations regarding these agents. Currently, a single-dose lapse is recommended; however, this may be inadequate given the unpredictable effects of these medications on gastric emptying and the extremely long half-lives of the most commonly used GLP-1 agonist medications. Point-of-care gastric ultrasound appears to be an inexpensive and effective modality that should be utilized for patients using these medications, when possible, to help guide clinical decision-making. Future research is extremely necessary to determine more evidence-based preoperative guidelines for these patients to mitigate risks.
